# Efficacy of SGLT2 inhibitors, GLP-1 receptor agonists, and aerobic exercise for moderate-to-severe obstructive sleep apnea in overweight or obese patients: a network meta-analysis

**DOI:** 10.3389/fendo.2026.1869479

**Published:** 2026-08-17

**Authors:** Jia Zheng, Zhiqi Zhu, Yu Bao, Yucai Hu, Zhengwei Dong, Anshe Zhao, Qiaozhi Li, Yongxia Wang, Mingjun Zhu, Peng Chen

**Affiliations:** 1 Department of Cardiovascular Disease, The First Affiliated Hospital of Henan University of Chinese Medicine, Zhengzhou, Henan, China; 2 Collaborative Innovation Center of Prevention and Treatment of Major Diseases by Chinese and Western Medicine, Zhengzhou, Henan, China

**Keywords:** aerobic exercise, GLP-1 receptor agonists, network meta-analysis, obstructive sleep apnea, SGLT2 inhibitors

## Abstract

**Objective:**

Obstructive sleep apnea (OSA) is a highly prevalent sleep disorder strongly linked to obesity and substantially increases the risk of cardiovascular and metabolic diseases. Sodium–glucose cotransporter 2 (SGLT2) inhibitors, glucagon-like peptide-1 (GLP-1) receptor agonists, and aerobic exercise have shown potential in improving OSA through distinct metabolic and physiological mechanisms. However, direct comparative evidence of their efficacy remains limited. This network meta-analysis aimed to compare the effects of SGLT2 inhibitors, GLP-1 receptor agonists, and aerobic exercise on OSA severity in overweight or obese patients.

**Methods:**

We searched four electronic databases, PubMed, EMBASE, the Cochrane Library, and Web of Science, for articles published before October 31, 2025, without language restrictions. The analysis primarily included randomized controlled trials (RCTs); a limited number of case–control studies were also incorporated due to the scarcity of direct comparative evidence. Primary efficacy outcomes were mean changes in the apnea–hypopnea index (AHI), body mass index (BMI), mean peripheral oxygen saturation (SpO_2_), and Epworth Sleepiness Scale (ESS) score. The certainty (confidence) of the evidence for every network estimate was appraised with the Confidence in Network Meta-Analysis (CINeMA) framework, which operationalizes the GRADE approach for network meta-analysis.

**Results:**

A total of 15 studies (13 RCTs and 2 case–control studies) involving 1,877 participants were included in the analysis. GLP-1 receptor agonists demonstrated the greatest reduction in AHI compared with placebo (mean difference [MD] = −15.28 events/h; 95% CI, −22.22 to −8.35). They also showed significant benefit versus placebo in lowering BMI (MD = −1.78 kg/m^2^; 95% CI, −2.15 to −1.41) and improving mean SpO_2_ (MD = 0.40%; 95% CI, 0.25 to 0.55). Although GLP-1 receptor agonists yielded a statistically significant improvement in ESS score versus placebo (MD = −0.20; 95% CI, −0.26 to −0.14), this effect was an order of magnitude below the 2-point minimal clinically important difference (MCID) for the ESS and is therefore not clinically meaningful. Aerobic exercise ranked highest in surface under the cumulative ranking curve (SUCRA) analysis for this outcome. No network estimate was rated as high certainty. Confidence was moderate for the effect of GLP-1 receptor agonists on BMI, low for their effects on AHI, mean SpO_2_, and ESS score versus placebo, and very low for all remaining comparisons, mainly because of within-study bias, imprecision, and suspected reporting bias.

**Conclusion:**

Compared with placebo, GLP-1 receptor agonists reduced AHI and BMI and improved mean SpO_2_, and ranked highest for these outcomes in the SUCRA analysis. Comparisons between the active interventions, however, were largely non-significant and rested on indirect evidence, and the certainty of the evidence was moderate at best, being low or very low for most comparisons. This treatment hierarchy should therefore be regarded as hypothesis-generating rather than as a basis for firm clinical recommendations. Within these limits, these findings suggest that GLP-1 receptor agonists may offer a promising therapeutic approach for managing OSA in overweight or obese patients with metabolic comorbidities, though this remains to be confirmed in larger, high-quality, head-to-head trials.

## Highlights

What is already known about this subject?

- SGLT2 inhibitors, GLP-1 receptor agonists, and aerobic exercise each individually reduce AHI and improve related outcomes in overweight/obese patients with OSA, mainly versus placebo or usual care.
- Direct head-to-head comparisons among these three interventions are lacking.

What are the new findings in your manuscript?

- This network meta-analysis is the first to simultaneously compare SGLT2 inhibitors, GLP-1 receptor agonists, and aerobic exercise for OSA, showing GLP-1RAs ranked highest for AHI reduction, BMI decrease, and mean SpO_2_ improvement.
- Aerobic exercise ranked highest for ESS score improvement, with all three interventions outperforming placebo on AHI.
- Certainty of the evidence, appraised with CINeMA, was moderate at best (GLP-1RAs versus placebo for BMI) and low or very low for all other comparisons, so the treatment hierarchy is hypothesis-generating.

How might your results change the direction of research or the focus of clinical practice?

- Results highlight GLP-1 receptor agonists as a promising pharmacotherapy for OSA in overweight/obese patients, prioritizing them in future head-to-head RCTs with long-term cardiovascular endpoints.
- Clinically, they support integrating GLP-1RAs (and aerobic exercise for symptomatic relief) into comprehensive OSA management beyond CPAP alone.

## Introduction

Obstructive sleep apnea (OSA) is a highly prevalent global sleep-related breathing disorder characterized by recurrent collapse of the upper airway during sleep, resulting in intermittent hypoxia, hypercapnia, and sleep fragmentation (1). An estimated 936 million adults worldwide are affected by OSA, with approximately 60% of cases closely associated with obesity (2). OSA not only causes excessive daytime sleepiness, impairs quality of life, and increases the risk of traffic accidents but also serves as an independent risk factor for a range of cardiovascular, cerebrovascular, and metabolic conditions, including hypertension, coronary artery disease, heart failure, stroke, insulin resistance, and type 2 diabetes (3, 4), constituting a major public health burden (5). Continuous positive airway pressure (CPAP) remains the traditional first-line therapy for OSA (6). Although CPAP effectively eliminates respiratory events and improves nocturnal oxygenation, long-term adherence is generally poor (7, 8). Moreover, as a purely mechanical ventilatory support modality, CPAP has limited impact on the core pathophysiological drivers of OSA, particularly obesity-related metabolic inflammation, fluid retention, and autonomic dysfunction (9, 10). Consequently, there is growing interest in comprehensive, sustainable intervention strategies that target the underlying mechanisms of OSA, a focus of both current clinical practice and research.

Given CPAP’s limitations in modifying OSA pathophysiology, attention has shifted toward pharmacological and lifestyle-based approaches that address the root causes of upper airway instability (11). The central role of obesity and its associated metabolic dysregulation in OSA pathogenesis has highlighted two novel drug classes, sodium–glucose cotransporter-2 (SGLT2) inhibitors and glucagon-like peptide-1 (GLP-1) receptor agonists, as particularly promising candidates (12, 13). SGLT2 inhibitors promote osmotic diuresis and urinary glucose excretion, leading to weight reduction and potentially attenuating nocturnal rostral fluid shift, thereby decreasing edema and collapsibility of peripharyngeal tissues (14). GLP-1 receptor agonists exert potent central appetite suppression and peripheral weight loss effects, directly reducing neck fat deposition (15); they may also confer additional benefits through anti-inflammatory actions and modulation of autonomic function. Recent clinical evidence, such as from the SURMOUNT-OSA trial, demonstrates that certain GLP-1 receptor agonists (e.g., tirzepatide) significantly reduce the apnea–hypopnea index (AHI) in OSA patients, with effects partially independent of weight loss (16). Concurrently, aerobic exercise, recognized as a cornerstone of lifestyle intervention, has been consistently shown in multiple systematic reviews to improve OSA severity and patient-reported symptoms through several mechanisms (17). These include enhanced function of upper airway dilator muscles, improved cardiopulmonary fitness, reduced systemic inflammation, and weight loss (18). Together, these pharmacological and lifestyle interventions represent complementary strategies that may act on distinct yet overlapping pathophysiological pathways in OSA.

Although SGLT2 inhibitors, GLP-1 receptor agonists, and aerobic exercise have each demonstrated therapeutic potential in overweight or obese OSA patients, existing evidence largely derives from individual studies comparing each intervention against placebo or standard care (19–21). Direct head-to-head randomized controlled trials (RCTs) comparing their relative efficacy are lacking. To address this gap, we conducted a network meta-analysis to systematically identify and synthesize all available clinical studies evaluating SGLT2 inhibitors, GLP-1 receptor agonists, and aerobic exercise for OSA treatment in this population. By integrating both direct and indirect comparisons, we aimed to assess the relative efficacy of these three intervention types in reducing AHI and BMI, improving Epworth Sleepiness Scale (ESS) scores, and increasing peripheral oxygen saturation (SpO_2_). This analysis seeks to establish an evidence-based hierarchy of treatments to inform clinical decision-making for overweight or obese patients with OSA.

## Methods

### Study registration and reporting standards

This study was reported in strict accordance with the extension of the Preferred Reporting Items for Systematic Reviews and Meta-Analyses (PRISMA) statement for network meta-analyses (PRISMA-NMA) (22). The protocol for this network meta-analysis was not registered in PROSPERO or in any other prospective registry of systematic reviews, and no protocol was published in advance. The eligibility criteria, the outcomes, and the analysis plan were nevertheless defined before the literature search was executed, and all prespecified outcomes are reported in this manuscript. We acknowledge the absence of prospective registration as a limitation, as it precludes independent verification that the review followed its intended plan.

### Selection criteria and study outcomes

We included parallel-group RCTs and case–control studies that met the following PICOS criteria:

#### Population

Adults aged ≥18 years diagnosed with moderate-to-severe OSA (AHI ≥15 events/hour) by polysomnography (PSG) or home sleep apnea testing, and with a baseline body mass index (BMI) ≥25 kg/m^2^ (classified as overweight or obese). To ensure baseline comparability, patients who were already receiving regular CPAP therapy before trial initiation were excluded.

#### Interventions

Treatment of OSA with SGLT2 inhibitors, GLP-1 receptor agonists, or aerobic exercise.

#### Comparators

Placebo or conventional therapy. Conventional therapy was defined as standardized basic management provided in addition to study interventions and, for this analysis, was limited to standard OSA education, including lifestyle recommendations such as weight loss advice, smoking cessation, alcohol reduction, and sleep hygiene guidance, but excluded structured exercise programs, formal calorie-restricted dietary plans (e.g., meal replacements or prescribed diets), and any device-based (e.g., CPAP, oral appliances) or surgical OSA treatments.

#### Outcomes

At least one of the prespecified outcomes of interest.

#### Study design

RCTs or case–control studies.

### Definition and measurement of outcome indicators

#### AHI

AHI is defined as the total number of apnea events (characterized by ≥90% reduction in oronasal airflow lasting ≥10 seconds) and hypopnea events (characterized by ≥30% reduction in airflow accompanied by either ≥3% oxygen desaturation or an electroencephalographic arousal) per hour of sleep, as measured by overnight PSG or validated home sleep apnea testing devices (23). In this analysis, moderate-to-severe OSA was defined as a baseline AHI ≥15 events/hour, consistent with the American Academy of Sleep Medicine (AASM) classification criteria (24). Only studies with a minimum follow-up duration of four weeks were eligible for inclusion.

#### ESS score

ESS score is a self-administered questionnaire with 8 items and a total score ranging from 0 to 24; higher scores indicate greater daytime sleepiness (25).

BMI: BMI is defined as weight in kilograms divided by the square of height in meters (kg/m^2^). It was measured by researchers using standardized procedures at baseline and post-intervention.

#### Mean SpO_2_

Mean SpO_2_ was defined as the arithmetic mean of the peripheral oxygen saturation recorded by pulse oximetry across the overnight sleep study, expressed as a percentage (%) (26, 27). Because the oxygenation indices reported in OSA trials (mean SpO_2_, minimum SpO_2_, the oxygen desaturation index, and T90) are distinct and not interchangeable (28), mean SpO_2_ was prespecified as the only oxygenation metric eligible for synthesis, and no pooling across different indices was performed.

### Literature search strategy

We systematically searched PubMed, Embase, the Cochrane Library, and Web of Science from database inception to October 31, 2025. The search strategy combined Medical Subject Headings (MeSH) terms and free-text keywords. English search terms included: “Obstructive Sleep Apnea,” “Sleep Apnea, Obstructive,” “GLP-1 Receptor Agonist,” “Liraglutide,” “Semaglutide,” “Tirzepatide,” “SGLT2 Inhibitor,” “Dapagliflozin,” “Empagliflozin,” “Canagliflozin,” “Exercise,” and “Aerobic exercise.” We also manually screened reference lists of included studies and relevant systematic reviews. The details of the search strategy conducted are presented in **Table 1**.

**Table 1:** Literature retrieval strategy.

Retrieval strategy	Term 1	Term 2	Term 3	Term 4
Subject word	Sodium-Glucose Transporter 2 Inhibitors	Glucagon-Like Peptide-1 Receptor Agonists	Exercise	Sleep Apnea, Obstructive
Free words	Sodium Glucose Transporter 2 Inhibitors SGLT-2 Inhibitors SGLT-2 Inhibitor Gliflozins Gliflozin Canagliflozin Dapagliflozin Empagliflozin Ertugliflozin Ipragliflozin Luseogliflozin Tofogliflozin	Glucagon Like Peptide 1 Receptor Agonists GLP-1 Receptor Agonists Incretin Mimetics GLP-1 Analogs GLP 1 Analogs Exenatide Lyxumia Liraglutide Saxenda Tanzeum Albiglutide Trulicity Dulaglutide Semaglutide Tirzepatide	Exercises training Endurance training Endurance exercise Resistance training Resistance exercise Strength training Strength exercise Physical activity Exercise intervention Training intervention Exercise-induced Trained-induced Aerobic exercise Isometric exercises Acute exercise Exercise training Physical exercise	Apneas, Obstructive Sleep Sleep Apneas, Obstructive Sleep Apnea Hypopnea Syndrome Obstructive Sleep Apnea Syndrome Obstructive Sleep Apnea Syndrome, Obstructive Sleep Apnea Syndrome, Sleep Apnea, Obstructive Sleep Apnea Syndrome, Obstructive OSAHS Upper Airway Resistance Sleep Apnea Syndrome Syndrome, Upper Airway Resistance, Sleep Apnea

Retrieval strategy: Term 1(Subject word OR Free words) And Term 4 (Subject word OR Free words); Term 2 (Subject word OR Free words) And Term 4. (Subject word OR Free words); Term 3 (Subject word OR Free words) And Term 4 (Subject word OR Free words).

### Study selection and data extraction

Two investigators independently conducted literature screening (title/abstract and full-text stages) and data extraction. Discrepancies were resolved through discussion or consultation with a third investigator. EndNote X9 software was used for reference management and duplicate removal. Data were extracted using a pre-designed, standardized form that captured the following information: first author, publication year, country, study design, sample size, baseline patient characteristics (age, AHI), intervention and comparator details (drug type and dose, exercise protocol), and follow-up duration.

### Risk of bias assessment

RCTs were assessed using the Cochrane risk-of-bias tool, and case–control studies were evaluated using the Newcastle–Ottawa Scale (NOS).

#### Cochrane risk assessment of RCT studies

Two independent reviewers applied the Cochrane tool to assess the methodological quality of included RCTs across seven domains: (1) random sequence generation, (2) allocation concealment, (3) blinding of participants and personnel, (4) blinding of outcome assessment, (5) incomplete outcome data, (6) selective reporting, and (7) other sources of bias; each domain was rated as low, high, or unclear risk of bias.

The same two reviewers used the NOS to evaluate case–control studies. The NOS comprises three domains, selection, comparability, and exposure, with a total score ranging from 0 to 9 stars; higher scores indicate better methodological quality. Under this semi-quantitative star system, all items except comparability are awarded up to 1 star, while comparability can receive up to 2 stars. Studies scoring at least 5 stars were considered eligible for inclusion in meta-analyses.

### Statistical analyses

We conducted a network meta-analysis to compare the effects of the different interventions, synthesizing direct and indirect evidence across the network. The network geometry of each outcome was displayed in a network plot. Analyses were performed in Stata/SE 17 with the mvmeta and network packages (commands network setup, network meta, network sidesplit and netfunnel). For each outcome we fitted a random-effects consistency model by restricted maximum likelihood, taking placebo as the reference. Because each study contributes several correlated treatment contrasts, the model is a multivariable rather than a multivariate one: the basic parameters are the mean differences (MDs) of each treatment versus placebo, and a common between-study variance (τ^2^) was assumed across contrasts. Multi-arm trials were handled by the data augmentation built into network setup. All outcomes were reported on the same scale, so MDs with 95% confidence intervals (CIs) were pooled directly and are reported for every contrast, together with a league table of all pairwise results. Treatments were ranked by their mean rank and by the surface under the cumulative ranking curve (SUCRA). We checked the consistency assumption globally, by comparing the consistency model with an unrelated-mean-effects (inconsistency) model, and locally in each closed loop, using node-splitting and the loop-specific method. Where a material source of bias was identified, a sensitivity analysis was carried out.

### Assessment of small study effects

To evaluate potential small-study effects and publication bias, we visually inspected comparison-adjusted funnel plots for outcomes supported by at least ten studies. For outcomes with fewer than ten studies, funnel plots were not generated, as they lack adequate power to reliably detect asymmetry in such cases.

### Assessment of the certainty of evidence

The certainty (confidence) of the evidence for every relative treatment effect obtained from the network was appraised using the Confidence in Network Meta-Analysis (CINeMA) framework, which adapts and operationalizes the GRADE approach for network meta-analysis (29–31). Assessments were performed in the CINeMA web application (https://cinema.ispm.unibe.ch/), which was supplied with the percentage contribution matrix derived from the network estimates generated in Stata/SE 17. Every pairwise comparison in each of the four networks was evaluated across six domains: (1) within-study bias, (2) reporting bias, (3) indirectness, (4) imprecision, (5) heterogeneity, and (6) incoherence. Each domain was rated as “no concerns”, “some concerns”, or “major concerns”.

Within-study bias was derived from the study-level Cochrane risk-of-bias judgements (RCTs) and Newcastle–Ottawa Scale scores (case–control studies), weighted by the percentage contribution of each study to the corresponding network estimate; comparisons to which non-randomized studies contributed a substantial proportion of the information were rated as raising major concerns, and the influence of unblinded outcome assessment was judged separately for objective outcomes (AHI, BMI, mean SpO_2_) and for the subjective, self-reported outcome (ESS score). Reporting bias was informed by the comparison-adjusted funnel plots, by the number of contributing studies, and by whether the contributing trials had been prospectively registered; for outcomes in which funnel plots could not be reliably interpreted (mean SpO_2_ and ESS score), reporting bias was classified as “suspected”. Indirectness was assessed by examining whether the populations, interventions, and comparators of the contributing studies matched the review question, and whether the transitivity assumption was plausible across the network.

Imprecision, heterogeneity, and incoherence were judged within a partially contextualized framework, in which 95% confidence intervals (imprecision) and 95% prediction intervals (heterogeneity) were compared with a prespecified range of equivalence bounded by the minimal important difference (MID) of each outcome: 5 events/h for AHI (32), 1 kg/m^2^ for BMI, 1% for mean nocturnal SpO_2_, and 2 points for the ESS score (33). An estimate whose confidence interval crossed the null was rated as raising major concerns for imprecision; one whose confidence interval crossed the MID but not the null was rated as raising some concerns. Incoherence was evaluated with the loop-specific approach and the side-splitting method. Because closed loops were present only in the AHI and BMI networks, incoherence could not arise in the mean SpO_2_ and ESS networks and was therefore recorded as not applicable for those two outcomes.

The six domain judgements were combined into an overall confidence rating of high, moderate, low, or very low. Ratings started at high confidence and were downgraded by one level for each domain judged to raise some concerns and by two levels for each domain judged to raise major concerns, with very low as the lowest possible rating (34). Two reviewers (ZZ and YB) independently completed the assessment, and disagreements were resolved through discussion with a third reviewer (PC).

## Results

### Study selection

**Figure 1:**
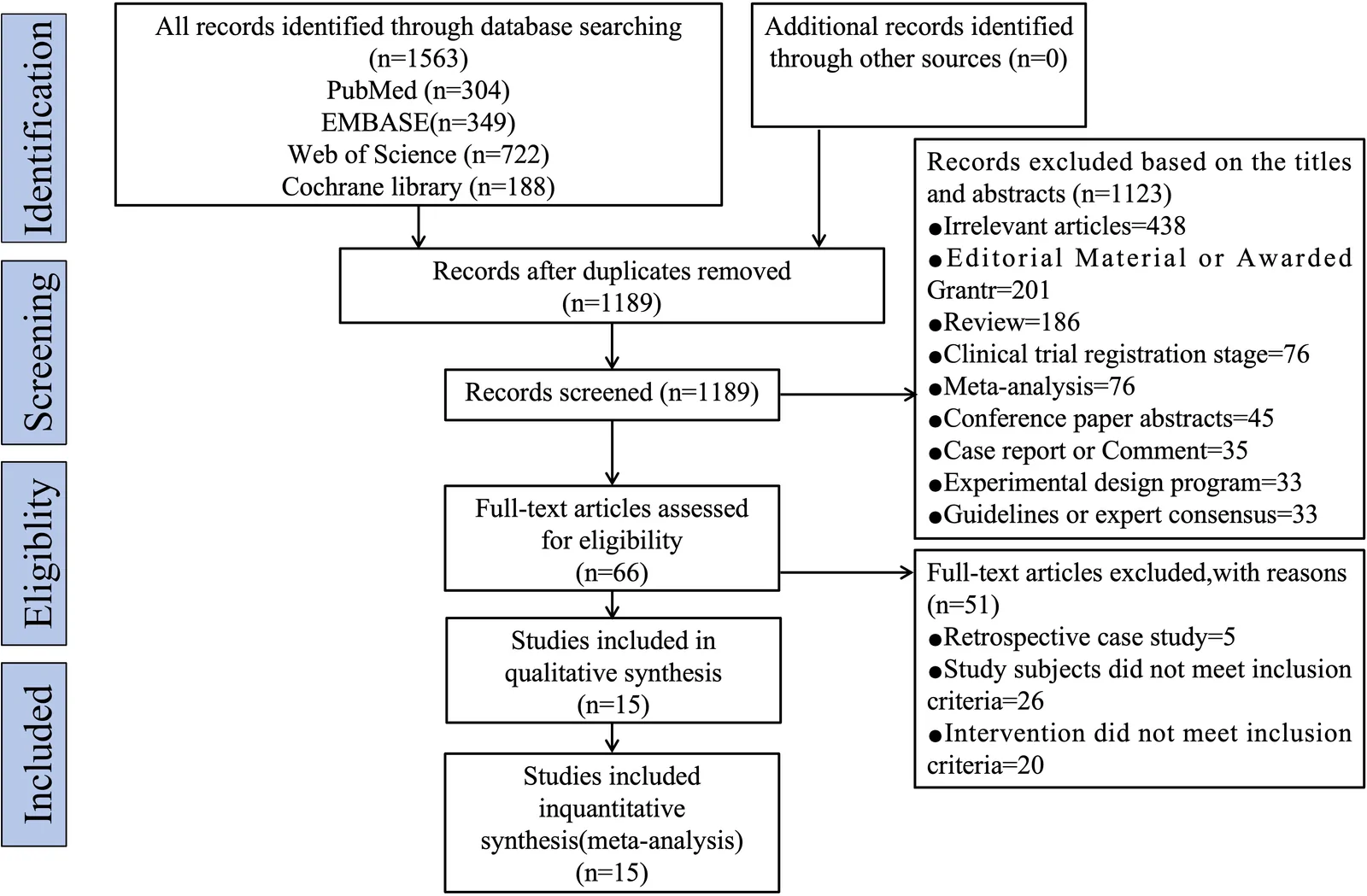
Literature search and selection.

The initial search of four databases yielded 1,563 records. After removing duplicates, 1,189 unique articles remained. Title and abstract screening excluded 1,123 studies, comprising 438 irrelevant articles, 186 reviews, 76 meta-analyses, 201 editorials or grant announcements, 27 comments, 8 case reports, 33 guidelines or expert consensus statements, 76 studies still in the clinical trial registration phase, 45 conference abstracts lacking sufficient data, and 33 study protocols. This left 66 articles for full-text assessment. Of these, 51 were excluded: 26 did not meet participant inclusion criteria, 5 were retrospective case studies, and 20 involved interventions that did not satisfy inclusion criteria. Ultimately, 15 studies were included in this network meta-analysis (**Figure 1**).

### Study characteristics

The review included 1,877 participants from nine countries and territories: seven studies were conducted in the Americas (United States, Brazil), six in Asia (China, Turkey), and two in Europe (Italy, Spain). Participant mean age ranged from 45 to 75 years, and trial sample sizes varied from 20 to 514. Nine trials enrolled participants with obesity, and five included those with overweight. All 15 studies reported AHI as a primary outcome. In all 15 included studies, AHI was measured by overnight polysomnography (PSG), and a hypopnea was defined as a ≥30% reduction in airflow accompanied by a ≥3% oxygen desaturation. Additionally, 10 studies reported BMI, 7 reported mean SpO_2_ levels, and 6 reported ESS score. Eight studies compared SGLT2 inhibitors, GLP-1 receptor agonists, or aerobic exercise against placebo, while seven compared these interventions with conventional treatment. Across the 15 trials, interventions included SGLT2 inhibitors (n = 3), GLP-1 receptor agonists (n = 5), conventional therapy (n = 7), and aerobic exercise (n = 7). Summary data for each included study are presented in **Table 2**.

**Table 2:** Characteristics of studies included in the systematic review.

Authors	Year	Country	Patients	Age	size	AHI	Interventions of experimental group	Interventions of control group	duration
Xie L, et al (35)	2024	China	Overweight	T:61.35±10.52 C:62.88±11.76	107	T: 25.62±6.10 C: 24.35±7.31	dapagliflozin 10 mg daily	Conventional glucose-lowering agents excluding SGLT2 inhibitors	3 months
Tang Y, et al (36)	2019	China	Overweight	T: 56.10 ± 7.21 C: 57.88 ± 10.07	36	T:37.45±6.04 C: 38.11±6.27	dapagliflozin 10 mg daily	Glimepiride 4 mg daily	24weeks
Armentaro G, et al (14)	2024	Italy	Overweight	T:76.2 ± 7.2 C:76.4 ± 6.8	514	T: 28.4±12.9 C: 26.0±13.6	dapagliflozin 10 mg daily	Conventional glucose-lowering agents excluding SGLT2 inhibitors	3 months
Malhotra A, et al (37)	2024	United States	Obesity	T: 47.3±11.0 C: 48.4±11.9	234	T: 52.9±30.5 C: 50.1±31.5	Tirzepatide 15 mg weekly	placebo	52weeks
Malhotra A, et al (38)	2024	United States	Obesity	T: 50.8±10.7 C: 52.7±11.3	235	T: 46.1±22.4 C: 53.1±30.2	Tirzepatide 15 mg weekly	placebo	52weeks
Blackman A, et al (39)	2016	United States	Obesity	T: 48.6±9.9 C: 48.4±9.5	334	T: 49.0±27.5 C: 49.3±27.5	Liraglutide 3.0 mg daily	placebo	28weeks
Jiang W, et al (40)	2023	China	Overweight	T: 55.7±7.4 C: 54.8±5.5	89	T: 31.0±7.3 C: 30.1±6.22	Liraglutide 0.6 mg or 1.2 mg daily	Conventional glucose-lowering agents excluding liraglutide	3 months
Amin RS, et al (41)	2015	United States	Obesity	46 ± 9	27	T: 50±32 C: 32.6±21	Liraglutide 1.8 mg daily	Conventional glucose-lowering agents excluding GLP-1 receptor agonists	26 weeks
Yang H, et al (42)	2018	China	Overweight	T: 46.3 ± 6.4 C: 48.6 ± 7.2	67	T: 20.2±7.5 C: 19.5±6.1	Aerobic exercise training (at the individualized heart rate corresponding to the ventilatory anaerobic threshold determined at baseline cardiopulmonary exercise testing) performed on a cycle ergometer, 30 min/session, 3 sessions/week, for 12 weeks.	maintaining usual lifestyle without specific exercise intervention	12 weeks
Jurado-García A, et al (43)	2020	Spain	Obesity	T: 52 ± 6.6 C: 50 ± 9.5	58	T: 29±20.8 C: 27±9.9	Progressive walking program (with intensity regulated according to the Borg Rating of Perceived Exertion scale), ≥5 days/week, for 6 months.	Pedometer-based monitoring of daily physical activity was recommended without additional structured exercise intervention.	6 months
Ueno-Pardi LM, et al (44)	2022	Brazil	Obesity	T: 53.0±7.0 C: 51±6.0	47	T: 45±29 C: 41±24	Supervised aerobic exercise (heart rate between the anaerobic threshold and the respiratory compensation point determined at cardiopulmonary exercise testing) performed on a stationary cycle ergometer,60 min/session, 3 sessions/week, for 6 months.	participants underwent routine follow-up and were instructed to refrain from regular exercise training	6 months
Lins-Filho O, et al (45)	2024	Brazil	Obesity	T: 51.2±2.5 C: 57.0±2.1	26	T: 55.1±7.1 C: 42.5±5.9	High-intensity interval training (90–95% of maximal heart rate) performed as running or brisk walking, 5 × 4-min bouts/session separated by 3-min active recovery intervals of low-intensity walking (50–55% of maximal heart rate), 3 sessions/week, for 12 weeks.	30 minutes of unsupervised physical activity daily	12weeks
Lins-Filho O, et al (46)	2023	Brazil	Obesity	T: 53.2±9.9 C: 55.1±9.8	38	T: 55.0±7.0 C: 41.4±5.9	High-intensity interval training (90–95% of maximal heart rate) performed as running or brisk walking, 5 × 4-min bouts/session separated by 3-min active recovery intervals of low-intensity walking (50–55% of maximal heart rate), 3 sessions/week, for 12 weeks.	30 minutes of unsupervised physical activity daily	12weeks
Mendelson M, et al (47)	2016	Turkey	Overweight	T: 54.40±6.57 C: 48.0±7.49	20	T:15.19±5.43 C:17.92±6.45	moderate-intensity training (60–70% of maximal oxygen uptake) performed on a cycle ergometer and a treadmill, 45–60 min/session, 3 sessions/week, for 12 weeks.	routine clinical management and advice provided	12weeks
Yilmaz Gokmen G, et al (48)	2019	Turkey	Obesity	T: 50.44±8.38 C:45.68±7.64	45	T:19.32±7.09 C:18.66±6.14	Tai Chi and Qigong training (at an intensity of 11–13 on the Borg Rating of Perceived Exertion scale), 60 min/session,3 sessions/week, for 12 weeks.	maintaining usual lifestyle without specific exercise intervention	12weeks

### Risk of bias within studies

**Figure 2:**
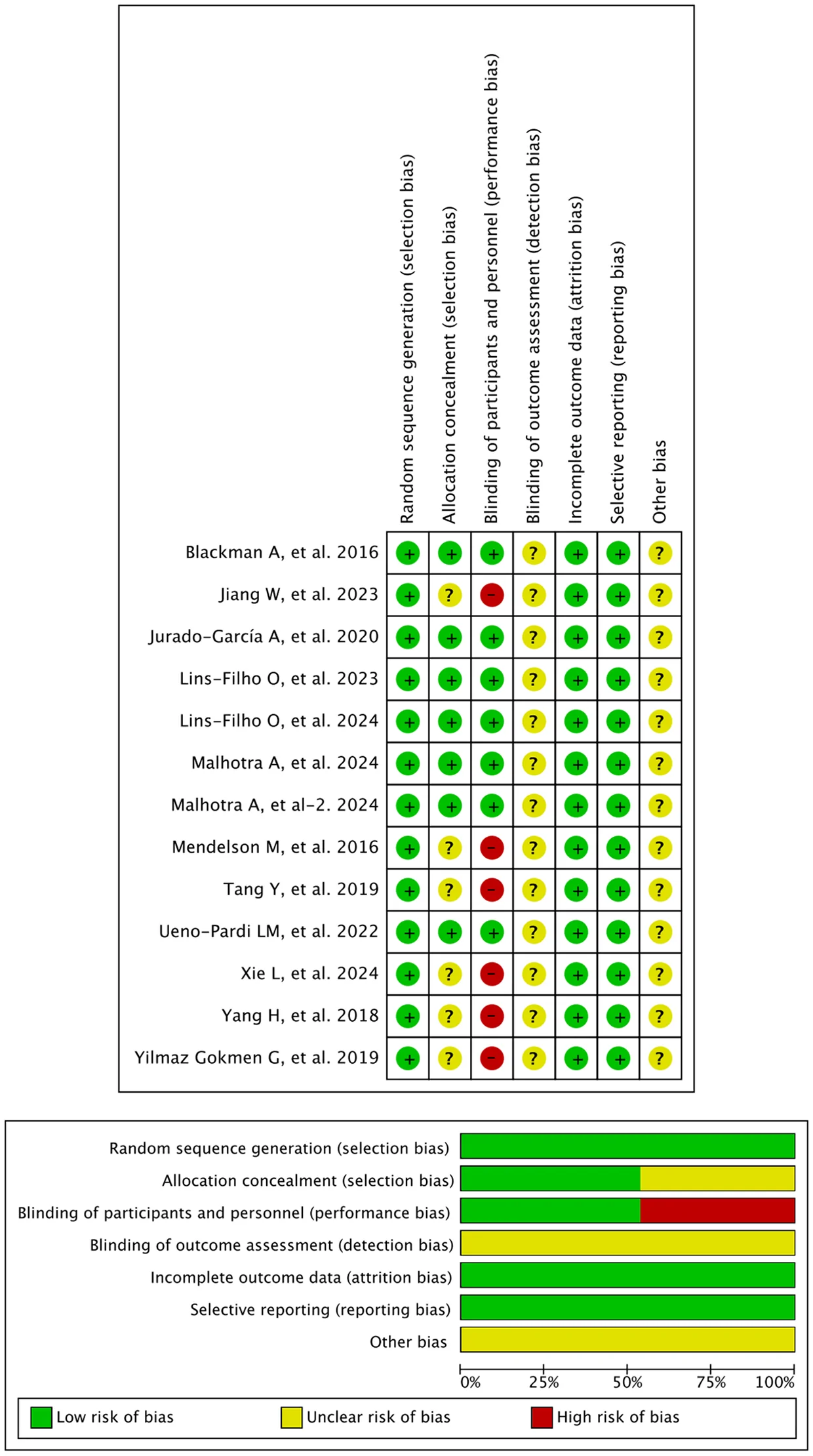
Risk of bias summary and graph.

Of the 15 included studies, two were case–control studies and 13 were RCTs. Among the RCTs, six were open-label and seven were double-blind. The six open-label RCTs showed a high risk of bias in the blinding of participants and personnel, whereas the risk related to the randomization process was low. However, allocation concealment and blinding of outcome assessors were unclear. In the seven double-blind RCTs, risks associated with randomization, allocation concealment, and blinding of participants and personnel were all low, but the risk concerning blinding of outcome assessors remained unclear. No study exhibited evidence of bias due to incomplete outcome data or selective reporting. Other potential biases were unclear across all included studies. The risk-of-bias summary and graph are presented in **Figure 2**.

The two case–control studies were evaluated using the NOS score. Both studies used a clear and appropriate diagnosis and definition of OSA; cases were enrolled consecutively; controls were drawn from the same community-based category as the cases but had not received treatment; baseline characteristics showed no statistically significant differences between the case and control groups; selection and analysis of controls were based on the key factor, the intervention, using identical statistical methods, ensuring group comparability; and exposure assessment relied on the same methodology (telephone calls or email) for both groups. Response rates were not reported for either group. Both studies received an overall NOS rating of 8 stars, indicating high methodological quality.

### Synthesis of results

#### Primary outcome

##### Differences in mean changes in AHI

**Figure 3:**
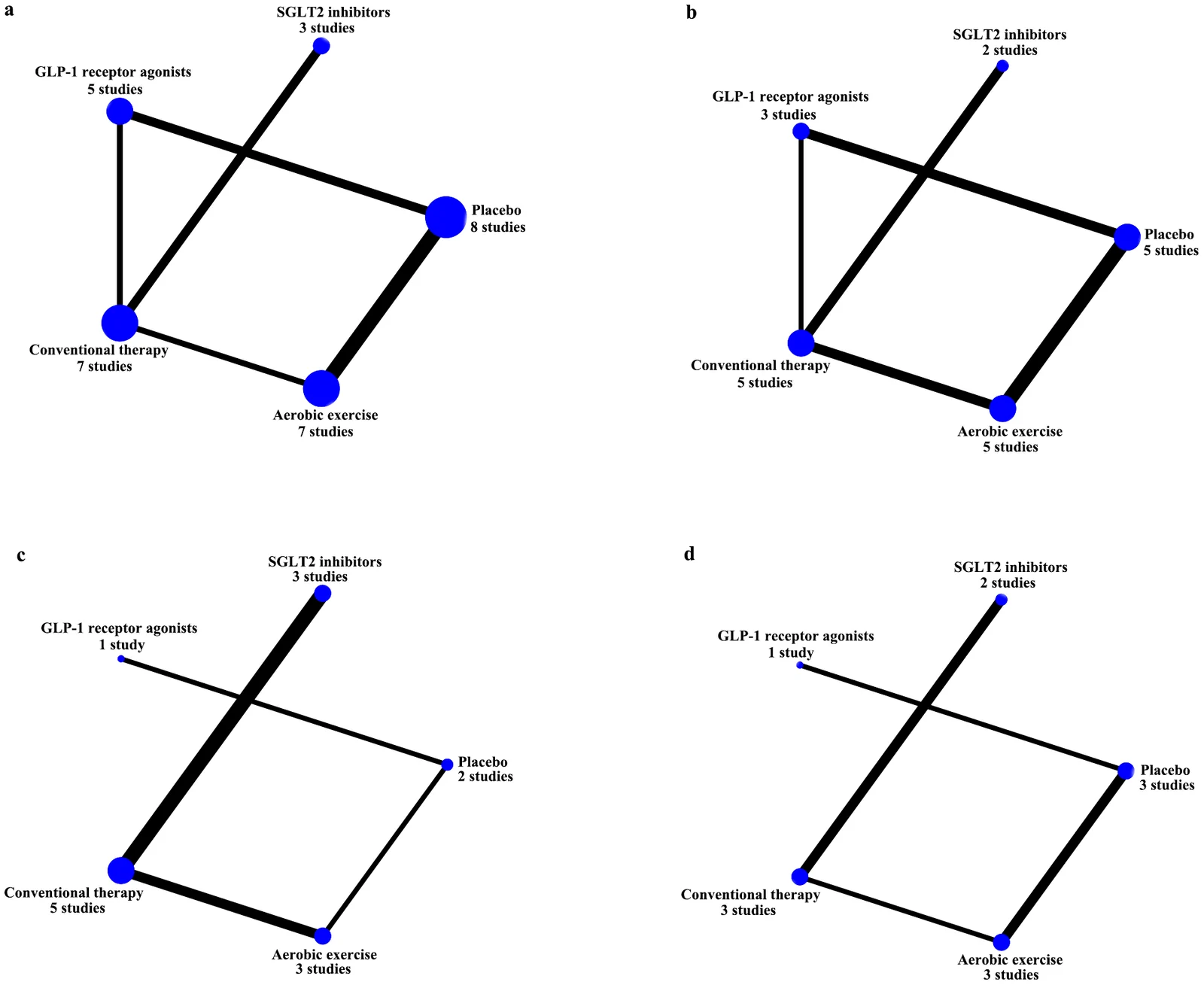
**(a)** Network plot for AHI. **(b)** Network plot for BMI. **(c)** Network plot for SpO2. **(d)** Network plot for ESS.

**Figure 4:**
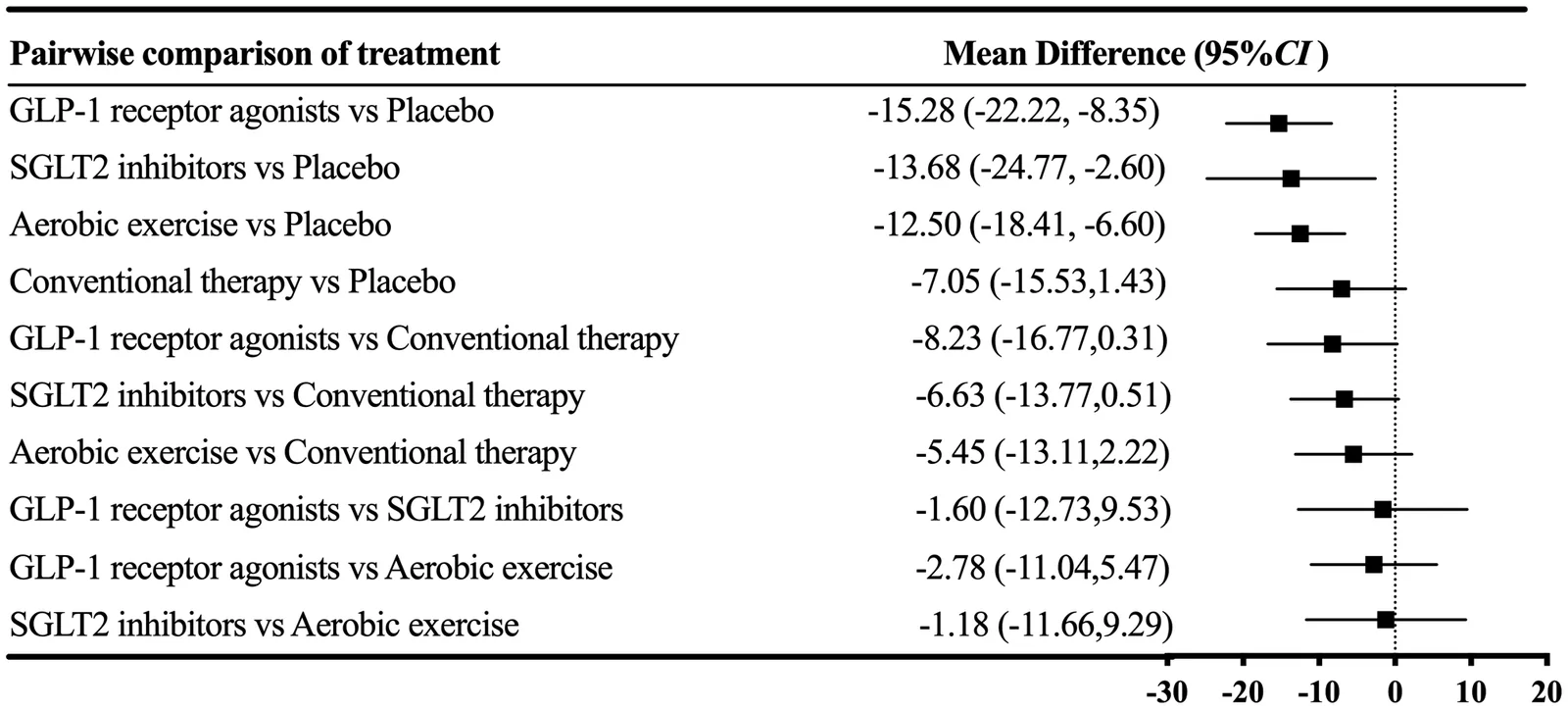
Forest plot of pairwise comparisons for AHI.

All 15 studies reported reductions in AHI across five intervention types. Network meta-analysis showed that the mean change in AHI was significantly greater with GLP-1 receptor agonists, SGLT2 inhibitors, and aerobic exercise than with placebo, as indicated by MDs below zero: MD = −15.28 events/h (95% CI −22.22 to −8.35), MD = −13.68 events/h (95% CI −24.77 to −2.60), and MD = −12.50 events/h (95% CI −18.41 to −6.60), respectively. No significant differences in mean AHI change were observed between any of these three interventions (SGLT2 inhibitors, GLP-1 receptor agonists, and aerobic exercise) and conventional therapy. Pairwise comparisons among the three active interventions also showed no statistically significant differences (**Figure 3a**, **Figure 4**, **Table 3a**).

**Table 3a:** Network meta-analysis of AHI.

GLP-1 receptor agonists				
-1.60 (-12.73,9.53)	SGLT2 inhibitors			
-2.78 (-11.04,5.47)	-1.18 (-11.66,9.29)	Aerobic exercise		
-8.23 (-16.77,0.31)	-6.63 (-13.77,0.51)	-5.45 (-13.11,2.22)	Conventional therapy	
-15.28 (-22.22, -8.35)	-13.68 (-24.77, -2.60)	-12.50 (-18.41, -6.60)	-7.05 (-15.53,1.43)	Placebo

Underlined values denote statistically significant comparisons, i.e., those whose 95 % confidence interval excludes zero.

#### Secondary outcomes

##### Differences in mean changes in BMI

Ten studies involving five therapies assessed changes in BMI. The network meta-analysis found that GLP-1 receptor agonists led to a significantly greater reduction in BMI compared with placebo (MD = −1.78 kg/m^2^; 95% CI −2.15 to −1.41). In contrast, neither SGLT2 inhibitors nor aerobic exercise differed significantly from placebo in their effect on BMI. Head-to-head comparisons showed that both GLP-1 receptor agonists and SGLT2 inhibitors were more effective than conventional therapy (MD = −2.55 kg/m^2^; 95% CI −4.21 to −0.90 and MD = −1.34 kg/m^2^; 95% CI −2.11 to −0.57, respectively). Additionally, GLP-1 receptor agonists demonstrated a significantly greater BMI reduction than aerobic exercise (MD = −1.35 kg/m^2^; 95% CI −2.41 to −0.29) (**Figure 3b**, **Table 3b**).

**Table 3b:** Network meta-analysis of BMI.

GLP-1 receptor agonists				
-1.21 (-3.04,0.61)	SGLT2 inhibitors			
-1.35 (-2.41, -0.29)	-0.14 (-1.68,1.39)	Aerobic exercise		
-1.78 (-2.15, -1.41)	-0.57 (-2.36,1.23)	-0.43 (-1.43,0.57)	Placebo	
-2.55 (-4.21, -0.90)	-1.34 (-2.11, -0.57)	-1.20 (-2.53,0.13)	-0.77 (-2.39,0.85)	Conventional therapy

Underlined values denote statistically significant comparisons, i.e., those whose 95 % confidence interval excludes zero.

##### Differences in mean changes in SpO_2_

Seven studies evaluating five therapies assessed changes in mean SpO2 levels. Network meta-analysis indicated that GLP-1 receptor agonists significantly improved mean SpO2 compared with placebo (MD = 0.40%; 95% CI 0.25 to 0.55). The other three interventions, SGLT2 inhibitors, aerobic exercise, and conventional therapy, did not differ significantly from placebo. When compared with conventional therapy, GLP-1 receptor agonists, SGLT2 inhibitors, and aerobic exercise all showed significantly greater increases in mean SpO2 (MD = 2.35%; 95% CI 0.16 to 4.53; MD = 2.60%; 95% CI 2.21 to 3.00; and MD = 1.95%; 95% CI 0.04 to 3.86, respectively). Pairwise comparisons among these three active interventions showed no statistically significant differences (**Figure 3c**, **Table 3c**).

**Table 3c:** Network meta-analysis of SpO_2_.

GLP-1 receptor agonists				
-0.25 (-2.48,1.97)	SGLT2 inhibitors			
0.40 (-0.66,1.46)	0.65 (-1.30,2.61)	Aerobic exercise		
0.40 (0.25,0.55)	0.65 (-1.56,2.87)	0.00 (-1.05,1.05)	Placebo	
2.35 (0.16,4.53)	2.60 (2.21,3.00)	1.95 (0.04,3.86)	1.95 (-0.23,4.13)	Conventional therapy

Underlined values denote statistically significant comparisons, i.e., those whose 95 % confidence interval excludes zero.

### Differences in mean changes in ESS score

Six studies involving five therapies were included in the analysis of ESS score reductions. Network meta-analysis showed that GLP-1 receptor agonists resulted in a significantly greater improvement in ESS score compared with placebo (MD = −0.20; 95% CI −0.26 to −0.14); the entire confidence interval for this effect lay far below the 2-point minimal clinically important difference (MCID) for the ESS in OSA (33), indicating that the improvement, although statistically significant, is not clinically meaningful. No significant differences were observed between SGLT2 inhibitors, aerobic exercise, conventional therapy, and placebo. However, head-to-head comparisons revealed that both SGLT2 inhibitors and aerobic exercise were significantly more effective than conventional therapy (MD = −2.11; 95% CI −3.31 to −0.91 and MD = −3.97; 95% CI −6.73 to −1.21, respectively) (**Figure 3d**, **Table 3d**). These were the only two comparisons whose point estimates reached or exceeded the MCID, although the confidence intervals for both also encompassed values below it.

**Table 3d:** Network meta-analysis of ESS.

Aerobic exercise				
-1.65 (-3.70,0.39)	GLP-1 receptor agonists			
-1.86 (-4.87,1.15)	-0.21 (-3.84,3.43)	SGLT2 inhibitors		
-1.85 (-3.89,0.19)	-0.20 (-0.26, -0.14)	0.01 (-3.63,3.64)	Placebo	
-3.97 (-6.73, -1.21)	-2.32 (-5.75,1.12)	-2.11 (-3.31, -0.91)	-2.12 (-5.55,1.32)	Conventional therapy

Underlined values denote statistically significant comparisons, i.e., those whose 95 % confidence interval excludes zero.

### Ranking probabilities

**Figure 5:**
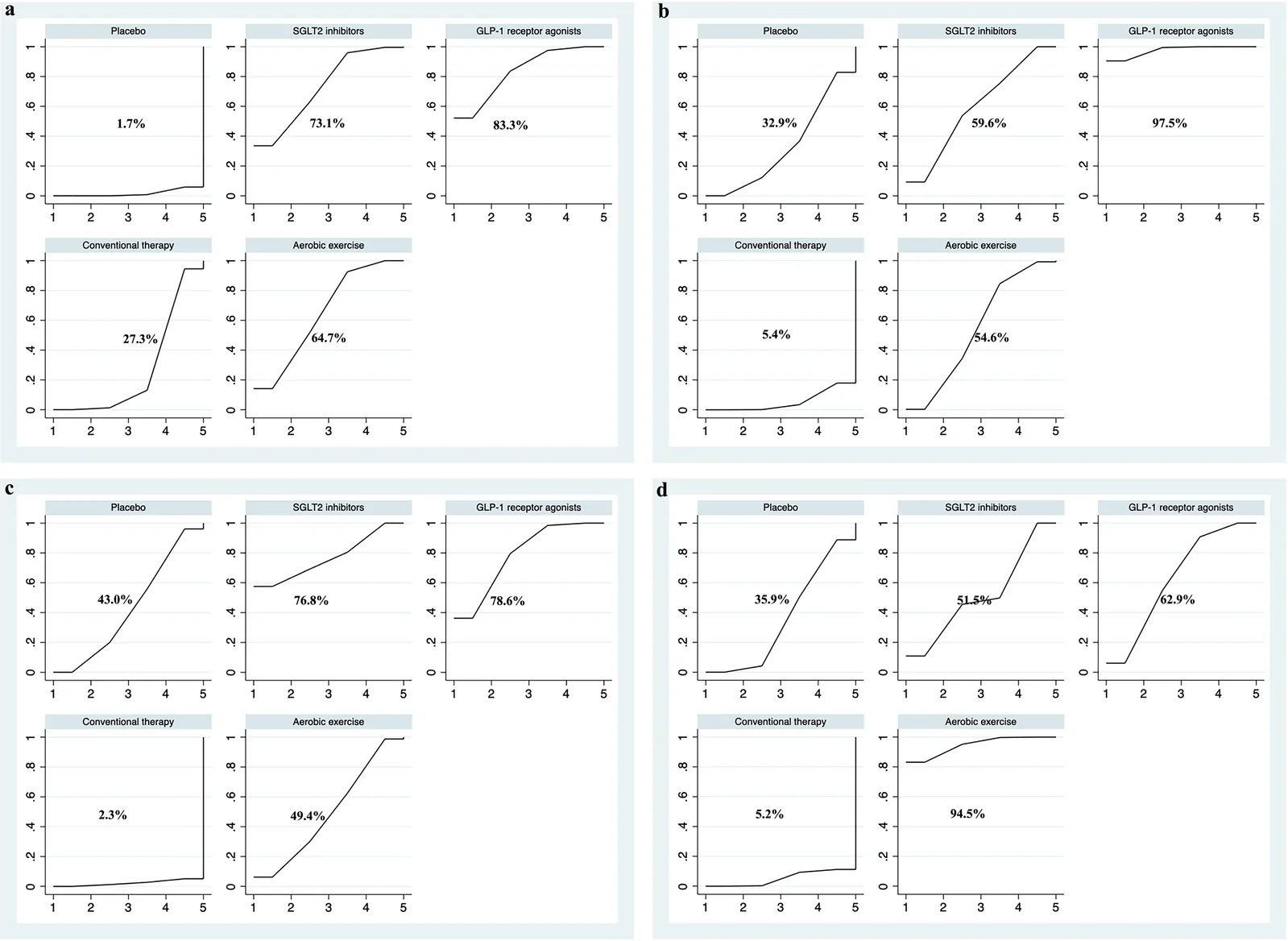
Ranking of treatment strategies based on probability of their protective effects on outcomes of **(a)** AHI, **(b)** BMI, **(c)** SpO2, **(d)** ESS according to the cumulative ranking area (SUCRA). Larger probability, stronger protective effects.

SUCRA values summarize the relative position of the four active therapies and placebo within the treatment hierarchy. GLP-1 receptor agonists ranked highest for reducing AHI, lowering BMI, and improving mean SpO2 levels, whereas aerobic exercise ranked highest for reducing ESS score. SUCRA values are presented in **Figure 5**. SUCRA values are calculated from the same network estimates as the pairwise comparisons reported above and carry no information about statistical significance or certainty; they can therefore be misleading when the evidence base is sparse and most contrasts are informed indirectly. In the present network only one comparison between active interventions attained statistical significance (GLP-1 receptor agonists versus aerobic exercise for BMI), and all remaining active-versus-active comparisons, for every outcome, were non-significant. This ranking should accordingly be read as a descriptive ordering of point estimates, not as evidence that any one active intervention is superior to another.

### Inconsistency test

Both AHI and BMI formed closed loops in the network, with inconsistency factor values of 1.49 and 2.87, respectively. The 95% confidence intervals for these inconsistency factors included zero, indicating no statistically significant inconsistency across studies for either outcome. Inconsistency test results are summarized in (**Supplementary Table 1**).

### Sensitivity analysis of AHI

A sensitivity analysis was performed by excluding the two case–control studies and repeating the network meta-analysis in the 13 RCTs, which still covered all five intervention nodes. The mean change in AHI remained significantly greater with GLP-1 receptor agonists, SGLT2 inhibitors, and aerobic exercise than with placebo: MD = −15.15 events/h (95% CI −22.57 to −7.74), MD = −14.65 events/h (95% CI −27.85 to −1.46), and MD = −12.58 events/h (95% CI −18.85 to −6.31), respectively. No significant differences were observed between any of the three active interventions and conventional therapy, and pairwise comparisons among the three active interventions again showed no statistically significant differences. In the SUCRA analysis, GLP-1 receptor agonists again ranked highest for AHI reduction. These results are consistent with the primary analysis in effect size, statistical significance, and treatment hierarchy, indicating that the AHI findings are robust to the exclusion of the non-randomized studies (**Supplementary Table 2**; **Supplementary Figure 2**).

### Risk of bias across studies

Comparison-adjusted funnel plots were used to evaluate potential small-study effects and publication bias for outcomes supported by at least ten studies. Of the four outcomes in this network meta-analysis, only AHI (15 studies) and BMI (10 studies) met this criterion and underwent funnel plot assessment. Visual inspection of the AHI funnel plot suggested mild asymmetry, while the BMI funnel plot appeared generally symmetric among studies with smaller standard errors (**Supplementary Figure 1**). These patterns indicate a possible minor degree of publication bias for both AHI and BMI. For mean SpO2 (7 studies) and ESS score (6 studies), the number of contributing studies was insufficient to allow reliable funnel plot interpretation; consequently, formal assessment of small-study effects was not conducted for these outcomes.

### Certainty of the evidence

Confidence in the evidence for the 40 pairwise comparisons across the four networks is summarized in **Supplementary Table 3**. No comparison was rated as high confidence: one comparison was rated moderate, four were rated low, and the remaining 35 were rated very low.

For the primary outcome, AHI, the comparison of GLP-1 receptor agonists with placebo was rated low confidence. The estimate was precise, with the entire 95% CI (−22.22 to −8.35 events/h) lying beyond the MID of 5 events/h, heterogeneity was low, and the network was coherent; the rating was downgraded for within-study bias (unclear allocation concealment and unclear blinding of outcome assessment in several contributing trials) and for suspected reporting bias (mild asymmetry of the comparison-adjusted funnel plot). Aerobic exercise versus placebo was rated very low confidence, with additional downgrading for heterogeneity arising from marked variation in exercise modality, intensity, session duration, and program length. SGLT2 inhibitors versus placebo was likewise rated very low confidence, because the two case–control studies contributed a large share of the information (major concerns for within-study bias) and because the 95% CI (−24.77 to −2.60 events/h) crossed the MID (some concerns for imprecision). All comparisons involving conventional therapy, and all comparisons among the three active interventions, were rated very low confidence, principally because their confidence intervals crossed the null (major concerns for imprecision) and because the active-versus-active estimates were informed exclusively by indirect evidence.

For BMI, GLP-1 receptor agonists versus placebo was the only estimate in the entire review to attain moderate confidence: the contributing trials were double-blind and at low risk of bias for this objective outcome, the estimate was precise (95% CI −2.15 to −1.41 kg/m^2^) and lay entirely beyond the MID of 1 kg/m^2^, heterogeneity was low, and the network was coherent; the rating was downgraded by a single level for suspected reporting bias. GLP-1 receptor agonists versus conventional therapy was rated low confidence, downgraded for suspected reporting bias and for imprecision, because the 95% CI (−4.21 to −0.90 kg/m^2^) crossed the MID. All remaining BMI comparisons, including SGLT2 inhibitors versus conventional therapy and GLP-1 receptor agonists versus aerobic exercise, were rated very low confidence.

For mean SpO_2_ and ESS score, no comparison exceeded low confidence. GLP-1 receptor agonists versus placebo was rated low confidence for both outcomes: although the pooled estimates were statistically significant and precise, they were clinically trivial, with the entire 95% CI lying inside the prespecified range of equivalence (mean SpO_2_: 0.25% to 0.55%, MID 1%; ESS score: −0.26 to −0.14 points, MID 2 points). Every other estimate for these two outcomes was rated very low confidence. Downgrading was driven by imprecision within these sparse networks (7 and 6 contributing studies, respectively), by within-study bias — a particular concern for the ESS score, a subjective outcome assessed in trials in which participants could not be blinded to aerobic exercise — and by reporting bias, which was classified as suspected because the small number of studies precluded funnel-plot evaluation. The apparent superiority of aerobic exercise over conventional therapy on the ESS score (MD = −3.97; 95% CI −6.73 to −1.21) was therefore supported by very low confidence evidence only.

Because SUCRA values are derived from the same network estimates, the treatment hierarchy presented above (**Figure 5**) inherits the confidence of the underlying pairwise comparisons and should be regarded as low to very low certainty evidence.

## Discussion

OSA is a globally prevalent sleep disorder closely linked to obesity and associated with substantially increased risks of cardiovascular and metabolic comorbidities (49). Although CPAP remains the standard first-line therapy, long-term adherence is often suboptimal and its effects on obesity-related pathophysiological alterations are limited (50). In recent years, two novel classes of glucose-lowering medications, SGLT2 inhibitors and GLP-1 receptor agonists, have shown potential therapeutic value in OSA management due to their established cardiometabolic benefits (51). Concurrently, aerobic exercise, as a foundational lifestyle intervention, ameliorates OSA through multiple mechanistic pathways (52). Nevertheless, high-level evidence directly comparing the efficacy of these three interventions remains scarce.

### Summary of key findings

This network meta-analysis systematically compared the efficacy of SGLT2 inhibitors, GLP-1 receptor agonists, and aerobic exercise in overweight or obese patients with OSA. All three interventions significantly outperformed placebo in improving AHI, a core metric of OSA severity, with GLP-1 receptor agonists ranking highest, although the pairwise comparisons among the three active interventions were not statistically significant for this outcome. Furthermore, GLP-1 receptor agonists ranked highest for reducing BMI and enhancing mean SpO_2_. In contrast, aerobic exercise ranked highest for improving ESS score. It must be emphasized, however, that the certainty of the evidence underpinning this hierarchy was moderate at best.

### Outcome-specific analysis

AHI is the most widely used and best-validated index for diagnosing and grading OSA severity, directly quantifying the frequency of respiratory events during sleep (53). It also represents the most objective metric for evaluating treatment effects on OSA pathophysiology (54). In our analysis, GLP-1 receptor agonists, SGLT2 inhibitors, and aerobic exercise all significantly reduced AHI compared with placebo. SUCRA ranking identified GLP-1 receptor agonists as the intervention with the highest probability of being the most effective; the pairwise comparisons among the three active interventions were not statistically significant, however, so this ranking does not establish that GLP-1 receptor agonists are superior to SGLT2 inhibitors or aerobic exercise for AHI. These agents produce substantial weight loss through central appetite suppression, thereby reducing neck fat deposition and airway collapsibility (37). Importantly, emerging evidence suggests additional weight-independent benefits, including anti-inflammatory effects and potential modulation of respiratory drive via central GLP-1 receptors (55, 56). SGLT2 inhibitors lower AHI primarily through osmotic diuresis, which reduces nocturnal fluid shift to the upper airway and decreases peripharyngeal edema, a weight-independent mechanism distinct from conventional approaches (57). Aerobic exercise improves OSA through multiple pathways: weight reduction, anti-inflammatory effects, enhanced upper airway muscle function, and improved autonomic balance (58–60). These reductions, however, should not be read as evidence of cure. Although the AHI reduction achieved by each intervention (−15.28, −13.68, and −12.50 events/h, respectively) comfortably exceeded the minimal clinically important difference of 5 events/h for the AHI (32), all included trials enrolled patients with moderate-to-severe OSA (baseline AHI ≥ 15 events/h), and mean baseline values were considerably higher, so that a mean reduction of 12 to 15 events/h left the average patient with a post-treatment AHI that remained at or above 15 events/h. The benefit of these therapies is real but incomplete: they attenuate disease severity without eliminating the disease.

BMI serves as an objective measure of obesity in OSA and a key outcome for evaluating obesity management. Our analysis showed that GLP-1 receptor agonists produced the largest reduction in BMI, significantly exceeding placebo, conventional therapy, and aerobic exercise; the comparison with SGLT2 inhibitors did not reach statistical significance. The contrast with aerobic exercise (MD = −1.35 kg/m^2^; 95% CI −2.41 to −0.29) was, in fact, the only comparison between active interventions to attain significance anywhere in this network, and it was rated very low certainty. This effect stems from central appetite suppression and peripheral metabolic actions, mechanisms well established in trials such as the STEP program (61). These include delayed gastric emptying, enhanced satiety, and altered lipid metabolism (62). SGLT2 inhibitors induce moderate weight loss primarily through osmotic diuresis and caloric excretion via glycosuria, though their effect size is generally smaller than that of GLP-1 receptor agonists (20). Aerobic exercise improves body composition by reducing fat mass and increasing lean mass, but its impact on total body weight is more modest and highly dependent on adherence and exercise intensity (17, 63). Notably, the magnitude of BMI reduction across interventions does not fully align with their efficacy in lowering AHI, indicating that weight loss is an important, but not exclusive, mechanism underlying OSA improvement.

Mean SpO_2_ is a critical indicator of intermittent hypoxia severity in OSA and directly reflects the efficacy of interventions in improving gas exchange (37). Our analysis revealed distinct response patterns: only GLP-1 receptor agonists showed statistically significant improvement in mean SpO_2_ compared with placebo, whereas both SGLT2 inhibitors and aerobic exercise demonstrated significant effects only when compared with conventional therapy. The underlying mechanisms parallel those outlined above for AHI: anti-inflammatory actions and possible modulation of respiratory control centers with GLP-1 receptor agonists (64); reduced peripharyngeal edema through fluid regulation with SGLT2 inhibitors, whose efficacy varies with individual fluid metabolism (65); and enhanced cardiopulmonary function, respiratory muscle endurance, and autonomic regulation with aerobic exercise (52). Notably, despite these differing mechanisms, all three interventions effectively alleviated OSA-related nocturnal hypoxemia in the included studies, underscoring their complementary roles in comprehensive OSA management.

The ESS score serves as a core indicator for assessing subjective symptoms in patients with OSA, and its improvement reflects the direct impact of treatment on quality of life (66). In this study, the improvement in ESS score with GLP-1 receptor agonists, although statistically significant, amounted to only 0.20 points, an order of magnitude below the 2-point MCID established for the ESS in patients with OSA (33). Interpreted against this benchmark, the intervention that produced the largest reductions in AHI and BMI in our network produced no clinically meaningful improvement in daytime sleepiness. Only aerobic exercise and SGLT2 inhibitors, and only when compared with conventional therapy, generated point estimates that reached or exceeded the MCID (−3.97 and −2.11 points, respectively), although their confidence intervals extended below it and neither differed significantly from placebo. The higher ranking of aerobic exercise for reducing ESS scores is mechanistically plausible: beyond decreasing respiratory events, it exerts multidimensional psychophysiological effects that may more directly alleviate the subjective experience of daytime sleepiness (17, 52). The relatively modest ESS improvements observed with pharmacological interventions align with the well-recognized phenomenon of residual sleepiness (67, 68). More broadly, the difficulty of achieving a clinically meaningful improvement in quality of life is one of the central unresolved problems in OSA therapeutics. The ESS is subject to floor effects: when mean baseline scores lie near or below the conventional threshold for excessive daytime sleepiness (>10 points), the scope for a 2-point improvement is arithmetically constrained. Future trials should therefore enroll patients with clinically significant sleepiness and report the proportion of participants achieving the MCID rather than the mean change alone. It should be noted that ESS, as a subjective scale, is susceptible to recall bias (69). Future studies should incorporate more objective measures of daytime function, such as the Multiple Sleep Latency Test, to validate these findings (70).

### Certainty of the evidence and implications for clinical recommendations

Confidence was constrained by three recurring limitations. First, within-study bias: several contributing trials were open-label with unclear allocation concealment, blinding of outcome assessment was rarely reported, and two non-randomized case–control studies contributed to the AHI, BMI, and mean SpO_2_ networks. Second, imprecision: with only 15 studies distributed across five nodes, most comparisons rested on modest numbers of participants, and the confidence intervals for all active-versus-active contrasts crossed the null. Third, reporting bias could not be excluded for AHI and BMI, and could not be evaluated at all for mean SpO_2_ and ESS score. In addition, the complete absence of head-to-head trials means that every comparison between GLP-1 receptor agonists, SGLT2 inhibitors, and aerobic exercise rests entirely on indirect evidence, and therefore on a transitivity assumption that cannot be verified empirically in a network of this size.

Within the GRADE framework, low or very low certainty evidence can support, at most, conditional (weak) recommendations, and cannot by itself justify a change in first-line practice (31, 34). Our findings should therefore be read as an evidence-based prioritization of hypotheses rather than as a definitive treatment ranking. The moderate-confidence estimate for BMI reduction with GLP-1 receptor agonists is the most secure finding of this review and accords with the large body of trial evidence in obesity; the low-confidence AHI estimate is directionally consistent with the SURMOUNT-OSA trial and is biologically plausible, although the magnitude of benefit remains uncertain. Conversely, the statistically significant but clinically trivial effects of GLP-1 receptor agonists on mean SpO_2_ and ESS score illustrate why statistical significance alone is an insufficient basis for clinical recommendation.

### Study limitations

To our knowledge, this is the first network meta-analysis to compare GLP-1 receptor agonists, SGLT2 inhibitors, and aerobic exercise in overweight and obese patients with OSA. Every network estimate was interpreted against a prespecified minimal important difference and formally appraised with CINeMA, so that the treatment hierarchy is reported together with its certainty. Several limitations nevertheless remain. First, moderate heterogeneity existed across studies in terms of intervention protocols (e.g., drug dosage, exercise intensity), follow-up duration, and patient characteristics. The conventional therapy comparator, whose content in each trial is detailed in **Table 2**, was a further source of heterogeneity. Because conventional therapy acts as a common comparator through which several indirect comparisons are estimated, this variability weakens the transitivity assumption and may bias the corresponding network estimates. Second, comparison-adjusted funnel plots showed mild asymmetry for AHI and BMI, suggesting possible publication bias for these outcomes and a potential overestimation of treatment efficacy. Third, the analysis focused primarily on intermediate-term physiological markers and questionnaire-based outcomes, without systematic assessment of long-term cardiovascular hard endpoints, safety events, or health economic indicators. Fourth, and most importantly for the interpretation of our results, the certainty of the evidence formally appraised with CINeMA was moderate for a single comparison (GLP-1 receptor agonists versus placebo for BMI), low for four comparisons, and very low for the remaining 35 comparisons, with no estimate reaching high confidence.

### Future research directions

Future research should prioritize the following areas. The highest priority is conducting large-scale, long-term, head-to-head randomized controlled trials that directly compare these interventions and include hard clinical endpoints. Additionally, integrating technologies such as polysomnographic microstructure analysis, advanced imaging (e.g., dynamic MRI), and molecular biology approaches could further clarify the specific pathophysiological mechanisms through which each intervention improves OSA. Investigating whether combination therapy, pairing GLP-1 receptor agonists or SGLT2 inhibitors with aerobic exercise, yields synergistic effects also represents a clinically valuable direction.

## Conclusion

This network meta-analysis suggests that, in overweight or obese patients with OSA, GLP-1 receptor agonists, SGLT2 inhibitors, and aerobic exercise may each reduce AHI relative to placebo. In the SUCRA analysis, GLP-1 receptor agonists ranked highest for AHI, BMI, and mean SpO_2_, whereas aerobic exercise ranked highest for ESS score. Because direct head-to-head evidence is lacking and comparisons between the active interventions were largely non-significant, these rankings do not establish the superiority of any one intervention. GLP-1 receptor agonists may nevertheless represent a promising intervention for overweight or obese patients with OSA, a possibility that requires confirmation in large-scale, head-to-head randomized controlled trials before definitive clinical recommendations can be made. Nevertheless, the certainty of this evidence, formally appraised with CINeMA, was moderate for only one comparison and low or very low for all others; the treatment hierarchy presented here should therefore be interpreted as hypothesis-generating and is not yet sufficient to support strong clinical recommendations. Equally important, the benefits observed were incomplete. Although the reductions in AHI exceeded the minimal clinically important difference of 5 events/h, the average patient across all three interventions retained an AHI at or above 15 events/h and therefore remained within the moderate-to-severe range after treatment. These interventions should accordingly be regarded as adjuncts that attenuate, but do not eliminate, OSA in overweight or obese patients, rather than as replacements for established therapies.

## Data Availability

The original contributions presented in the study are included in the article/**Supplementary Material**. Further inquiries can be directed to the corresponding authors.

## Supplementary material

Supplementary Figure 1 Funnel plot of each outcome.

Supplementary Figure 2 Sensitivity Analysis of AHI.

Supplementary Table 1 Results of inconsistency testing for AHI and BMI.

Supplementary Table 2 Sensitivity Analysis of AHI.

Supplementary Table 3 Confidence in the evidence (CINeMA) for all pairwise comparisons in the AHI, BMI, SpO_2_, and ESS score networks.
