# Supplementary material for: Efficacy of SGLT2 inhibitors, GLP-1 receptor agonists, and aerobic exercise for moderate-to-severe obstructive sleep apnea in overweight or obese patients: a network meta-analysis
Source: Front Endocrinol (Lausanne). 2026 Aug 17;17:1869479. doi: 10.3389/fendo.2026.1869479 (PMC13526671; doi:10.3389/fendo.2026.1869479)
Supplement: Supplementary file 1 [file Table1.docx]

| Table 4 Results of inconsistency testing for AHI and BMI | | | |
| --- | --- | --- | --- |
| Outcome indicators | Closed loop | IF | 95%CI |
| AHI | Placebo- GLP-1 receptor agonists-Conventional hypoglycemic therapy-Aerobic exercise | 1.49 | 0.00-22.13 |
| BMI | Placebo- GLP-1 receptor agonists-Conventional hypoglycemic therapy-Aerobic exercise | 2.87 | 0.00-8.29 |
