# Supplementary material for: Efficacy of SGLT2 inhibitors, GLP-1 receptor agonists, and aerobic exercise for moderate-to-severe obstructive sleep apnea in overweight or obese patients: a network meta-analysis
Source: Front Endocrinol (Lausanne). 2026 Aug 17;17:1869479. doi: 10.3389/fendo.2026.1869479 (PMC13526671; doi:10.3389/fendo.2026.1869479)
Supplement: Supplementary file 2 [file Table2.docx]

| Supplementary Table 2 Sensitivity Analysis of AHI | | | | |
| --- | --- | --- | --- | --- |
| GLP-1 receptor agonists |  |  |  |  |
| -0.50 (-13.99,12.98) | SGLT2 inhibitors |  |  |  |
| -2.58 (-11.49, 6.34) | -2.08 (-14.59,10.44) | Aerobic exercise |  |  |
| -7.67 (-17.37, 2.02) | -7.17 (-16.54,2.20) | -5.10 (-13.40,3.20) | Conventional therapy |  |
| -15.15 (-22.57, -7.74) | -14.65 (-27.85, -1.46) | -12.58 (-18.85, -6.31) | -7.48 (-16.77,1.81) | Placebo |
