# Supplementary material for: Efficacy of SGLT2 inhibitors, GLP-1 receptor agonists, and aerobic exercise for moderate-to-severe obstructive sleep apnea in overweight or obese patients: a network meta-analysis
Source: Front Endocrinol (Lausanne). 2026 Aug 17;17:1869479. doi: 10.3389/fendo.2026.1869479 (PMC13526671; doi:10.3389/fendo.2026.1869479)
Supplement: Supplementary file 3 [file Table3.docx]

Supplementary Table 3. Confidence in the evidence (CINeMA) for all pairwise comparisons in the AHI, BMI, mean SpO₂, and ESS score networks.

| **Comparison** | **Within-study bias** | **Reporting bias** | **Indirectness** | **Imprecision** | **Heterogeneity** | **Incoherence** | **Confidence rating** |
| --- | --- | --- | --- | --- | --- | --- | --- |
| **Outcome: apnea–hypopnea index (AHI)** | | | | | | | |
| GLP-1RA vs placebo | Some | Some | No | No | No | No | Low |
| SGLT2i vs placebo | Major | Some | No | Some | No | No | Very low |
| AE vs placebo | Some | Some | No | No | Some | No | Very low |
| CT vs placebo | Some | Some | No | Major | Some | No | Very low |
| GLP-1RA vs CT | Some | Some | No | Major | No | No | Very low |
| SGLT2i vs CT | Major | Some | No | Major | No | No | Very low |
| AE vs CT | Some | Some | No | Major | Some | No | Very low |
| GLP-1RA vs SGLT2i | Major | Some | Some | Major | No | No | Very low |
| GLP-1RA vs AE | Some | Some | Some | Major | Some | No | Very low |
| SGLT2i vs AE | Major | Some | Some | Major | Some | No | Very low |
| **Outcome: body mass index (BMI)** | | | | | | | |
| GLP-1RA vs placebo | No | Some | No | No | No | No | Moderate |
| SGLT2i vs placebo | Major | Some | No | Major | No | No | Very low |
| AE vs placebo | Some | Some | No | Major | Some | No | Very low |
| CT vs placebo | Some | Some | No | Major | Some | No | Very low |
| GLP-1RA vs CT | No | Some | No | Some | No | No | Low |
| SGLT2i vs CT | Major | Some | No | Some | No | No | Very low |
| AE vs CT | Some | Some | No | Major | Some | No | Very low |
| GLP-1RA vs SGLT2i | Major | Some | Some | Major | No | No | Very low |
| GLP-1RA vs AE | Some | Some | Some | Some | Some | No | Very low |
| SGLT2i vs AE | Major | Some | Some | Major | Some | No | Very low |
| **Outcome: peripheral oxygen saturation (SpO₂)** | | | | | | | |
| GLP-1RA vs placebo | Some | Some | No | No | No | NA | Low |
| SGLT2i vs placebo | Major | Some | No | Major | No | NA | Very low |
| AE vs placebo | Some | Some | No | Major | Some | NA | Very low |
| CT vs placebo | Some | Some | No | Major | Some | NA | Very low |
| GLP-1RA vs CT | Some | Some | No | Some | No | NA | Very low |
| SGLT2i vs CT | Major | Some | No | No | No | NA | Very low |
| AE vs CT | Some | Some | No | Some | Some | NA | Very low |
| GLP-1RA vs SGLT2i | Major | Some | Some | Major | No | NA | Very low |
| GLP-1RA vs AE | Some | Some | Some | Major | Some | NA | Very low |
| SGLT2i vs AE | Major | Some | Some | Major | Some | NA | Very low |
| **Outcome: Epworth Sleepiness Scale (ESS) score** | | | | | | | |
| GLP-1RA vs placebo | Some | Some | No | No | No | NA | Low |
| SGLT2i vs placebo | Major | Some | No | Major | No | NA | Very low |
| AE vs placebo | Major | Some | No | Major | Some | NA | Very low |
| CT vs placebo | Some | Some | No | Major | Some | NA | Very low |
| GLP-1RA vs CT | Some | Some | No | Major | No | NA | Very low |
| SGLT2i vs CT | Major | Some | No | Some | No | NA | Very low |
| AE vs CT | Major | Some | No | Some | Some | NA | Very low |
| GLP-1RA vs SGLT2i | Major | Some | Some | Major | No | NA | Very low |
| GLP-1RA vs AE | Major | Some | Some | Major | Some | NA | Very low |
| SGLT2i vs AE | Major | Some | Some | Major | Some | NA | Very low |

Domain judgements: "No" = no concerns; "Some" = some concerns; "Major" = major concerns; "NA" = not applicable (the SpO₂ and ESS score networks contained no closed loops, so incoherence could not arise). Overall confidence was rated as high, moderate, low, or very low, starting from high and downgrading one level for each domain rated "some concerns" and two levels for each domain rated "major concerns". Minimal important differences used to judge imprecision and heterogeneity: AHI, 5 events/h; BMI, 1 kg/m²; SpO₂, 1%; ESS score, 2 points. AE, aerobic exercise; AHI, apnea–hypopnea index; BMI, body mass index; CINeMA, Confidence in Network Meta-Analysis; CT, conventional therapy; ESS, Epworth Sleepiness Scale; GLP-1RA, glucagon-like peptide-1 receptor agonist; SGLT2i, sodium–glucose cotransporter 2 inhibitor; SpO₂, peripheral oxygen saturation.
